# Fermentation of Danggui Buxue Tang, an ancient Chinese herbal mixture, together with *Lactobacillus plantarum* enhances the anti-diabetic functions of herbal product

**DOI:** 10.1186/s13020-020-00379-x

**Published:** 2020-09-11

**Authors:** Rui Guo, Shuchen Guo, Xiong Gao, Huaiyou Wang, Weihui Hu, Ran Duan, Tina T. X. Dong, Karl W. K. Tsim

**Affiliations:** 1 Shenzhen Key Laboratory of Edible and Medicinal Bioresources, HKUST Shenzhen Research Institute, Shenzhen, 518057 China; 2 Key Laboratory of Innovative Drug for the Treatment of Serious Diseases Basing on the Chronic Inflammation, Shanxi University of Chinese Medicine, 121 Daxue Road, Yuci District, Jinzhong, 030619 China; 3 Division of Life Science and Center for Chinese Medicine, The Hong Kong University of Science and Technology, Hong Kong, China

**Keywords:** Danggui buxue tang, Fermentation, Bioconversion, Anti-diabetic functions

## Abstract

**Background:**

Danggui Buxue Tang (DBT), an ancient Chinese herbal decoction containing Astragali Radix and Angelicae Sinensis Radix at a ratio of 5: 1, is prescribed for menopausal women. Flavonoids and its flavonoid glycosides are considered as the major active ingredients within the herbal decoction; however, their amount is not controllable during the preparation. Besides, the aglycons within DBT are believed to have better gut absorption and pharmacological efficacy.

**Methods:**

The herbal extract of DBT was fermented with *Lactobacillus plantarum*. The amounts of flavonoid glucosides and its aglycones in the fermented product were analyzed by using UPLC-MS/MS. In addition, in vitro assays were employed to evaluate the efficacy of the fermented DBT in regulating the activities of α-glucosidase, α-amylase and lipase, as well as their antioxidant capacity (DPPH and T-AOC assays) and anti-glycation property (BSA-methylglyoxal, BSA-fructose, and arginine-methylglyoxal models).

**Results:**

The fermentation of DBT with *L. plantarum* drove a completed conversion of calycosin-7-O-β-D-glucoside and ononin to calycosin and formononetin, respectively. The chemical transformation could be probably mediated by β-glycosidase within the fermented product. Several in vitro assays corresponding to anti-diabetic functions were compared between parental DBT against its fermented product, which included the activities against α-glucosidase, α-amylase and lipase, as well as anti-oxidation and anti-glycation. The fermented DBT showed increased activities in inhibiting α-glycosidase, suppressing DPPH radical-scavenging and anti-glycation, as compared to the original herbal product.

**Conclusion:**

These results suggested that DBT being fermented with the probiotic *L. plantarum* could pave a new direction for fermentation of herbal extract, as to strengthen its pharmacological properties in providing health benefits.

## Background

Type 2 diabetes mellitus (T2DM) is a chronically metabolic syndrome characterized by insulin resistance and pancreatic β cell dysfunction, caused by inherited and/or environmental factors [1]. The global burden of diabetes is increasing worldwide, and the number of diabetic patients is expected to rise to 578 million by 2030 and to 700 million by 2045 [2]. Thus, an effective control of blood glucose is the key to prevent complication. Currently, many medicines are listed on the market to treat diabetes, including insulin, metformin, α-glucosidase inhibitors, thiazolidinediones and sodium-glucose transport protein 2 [3]; however, these treatments are leading to possible side effects, e.g. gastrointestinal discomfort and hypoglycemia [4, 5].

Traditional Chinese medicine (TCM) having less side-effect and irritation in general has been proposed in treating diabetic patients [6]. In particular, the formulated herbal mixtures have been commonly used in clinics for medical treatment. Among thousands of herbal formulae of TCM, Danggui Buxue Tang (DBT) is one of the simplest. DBT was first described in <  < *Neiwaishang Bianhuo Lun* >  > by Li Dongyuan in AD 1247 in China. He described DBT should have: Astragali Radix (AR; roots of *Astragalus memebranaceus* (Fisch.) Bunge var. *mongholicus* (Bunge) Hsiao) and Angelicae Sinensis Radix (ASR; roots of *Angelica sinensis* Oliv.) in 5 to 1 ratio. This herbal mixture has been utilized for nourishing “Qi” and enriching “Blood” for women suffering from menopausal symptoms. Pharmacologically, DBT is able to mitigate menopausal symptoms [7–10], to stimulate immune responses [11] and to accelerate bone regeneration [12, 13]. In addition, DBT has been found to alleviate insulin resistance and to relieve diabetic complication [14–16].

Calycosin-7-O-β-D-glucoside and ononin (a 7-O-β-D-glucopyranoside of formononetin) are two major flavonoid glucosides in DBT [17], and which are reported to have potential efficacy associated with diabetes [18]. The pharmacological efficacy of flavonoid glucosides is usually ascribed to their corresponding aglycones that are absorbed much easier than their glycoside forms [19, 20]. The de-glycosylation, triggered by β-glucosidase located in the human small intestine, is regarded as a critical process in improving metabolism of flavonoid glucosides [21, 22]. As a result, the aglycones can be absorbed effectively into blood circulation in attributing anti-diabetic functions [23].

In order to deglycosylate flavonoid glucosides to their corresponding aglycones, as well as to increase intestinal absorption of bioactive compounds, we adopt a bio-conversion method in vitro by means of microbial fermentation in a herbal mixture. Indeed, microorganisms have been employed in fermenting TCM, e.g. probiotic was used to ferment Scutellaria Radix [24], Atractylodis Macrocephalae Rhizoma [25] and red ginseng [26]. To improve the efficacy of DBT in fighting against diabetes, we developed a fermentation process of herbal extract together with *Lactobacillus plantarum*, a Gram-positive *Lactobacillus*, commonly found in fermented food products [27]. Thereafter, we compared the activities of fermented product against its parental herbal extract, which included: (i) the amounts of flavonoid glucosides and its aglycones; and (ii) the inhibitory activities of α-glycosidase, α-amylase, pancreatic lipase, antioxidant capacity and non-enzymatic glycation.

## Materials and methods

### Chemicals and reagents

Standards of calycosin-7-O-β-D-glucoside, ononin, calycosin, formononetin and rutin (Internal standard, IS) were supplied by Testing Laboratory for Chinese Medicine of HKUST (Hong Kong, China). The purity of each standard was > 98%, as detected by HPLC-DAD and ^13^C-NMR analysis. The HPLC grade acetonitrile and formic acid were obtained from Merck (Darmstadt, Germany). Deionized water (18 MW/cm) was supplied with a Direct-Q water purification system (Millipore, Milford, MA). Acquity UPLC C_18_ column (Waters, Milford, MA). Tris, p-nitrophenol (PNP), p-nitropheny-β-D-glucopyranoside (PNP-D-Glu), α-glucosidase, p-nitrophenyl-α-D-glucopyranoside, bovine serum albumin (BSA) and O-phenylenediamine were purchased from Macklin Biochemical (Shanghai, China). DPPH was gained from TCI Chemical Industry (Shanghai, China). Other materials were obtained from Sigma-Aldrich (St. Louis, MO).

### Preparation of herbal decoction

The roots of three-year-old *A. memebranaceus* var. *mongholicus* (Astragali Radix; Huangqi; AR) from Shanxi Province [28] and two-year-old *A. sinensis* roots (Angelicae Sinensis Radix; Danggui; ASR) from Minxian of Gansu Province [29] were collected in 2019. The herbs were identified morphologically by Dr. Tina TX Dong. The voucher specimens of AR (Lot: 20190320) and ASR (Lot: 20190412) were recorded in HKUST Shenzhen Research Institute. In preparing DBT, AR and ASR were weighed according to a ratio of 5:1 and then mixed well. The mixture was boiled in 8 volumes of water (v/w) for 2 h, and the extraction was repeated twice [12]. The extracts were dried by lyophilization and stored at − 80 °C. The chemical analysis of fermented DBT was carried out as described [8].

### Fermentation of DBT extract with *L. plantarum*

*L. plantarum* (GDM 1.191), a facultative heterofermentative lactic acid bacteria, was purchased from Guangdong Microbial Culture Collection Center (ACCC11095; Guangdong, China). *L. plantarum* is commonly found in most fermented foods [30]. The culture was inoculated twice in MRS broth (10 g peptone, 8.0 g lab-lemco’ powder, 4.0 g yeast extract, 20 g glucose, 2.0 g di-potassium hydrogen phosphate, 2.0 g tri-ammonium citrate, 5.0 g sodium acetate with 3 H_2_O, 0.2 g magnesium sulphate, 0.04 g manganese sulphate with 4 H_2_O, and 1 mL Tween in 80 L of water, pH 5.7 ± 0.2; from Hopebio, Qingdao, China) at 37 °C in anaerobic atmosphere (10% H_2_, 10% CO_2_, 80% N_2_) for 24 h to obtain the strain at end of exponential phase. A stock solution of DBT herbal extract was sterilized with a filter, and which was diluted with MRS medium. The inoculation of *L. plantarum* was adjusted to a concentration of 1 × 10^8^ CFU/mL, and the fermentation was performed at 37 °C under anaerobic condition, shaking in 100 rpm, until the late stationary phase. The growth of *L. plantarum* was determined by absorbance at 595 nm.

### UPLC-MS/MS analysis

The stock solutions of calycosin-7-O-β-D-glucoside, ononin, calycosin and formononetin were freshly prepared in methanol at 1 mg/mL. Mixed stock solution (200 μg/mL each) was prepared. Rutin (IS) at 20 μg/mL was diluted from the stock in methanol. The working standard solutions (0.092–200 μg/mL) for analytes were prepared by a serial diluent of mixed stock solution with methanol. The calibration standard solutions (0.023–50 μg/mL) for analytes were prepared by spiking an appropriate amount of working standard solutions into 150 μL blank matrix. The QC concentrations of tested samples were selected in 0.068, 1.84, and 16.6 μg/mL, respectively, at low, medium and high levels. The sample after fermentation (200 μL) and IS (50 μL) were shaken with vortex for 30 s. Then, adding 800 μL methanol to the mixture, vortexed for 2 min and centrifuged at 10,000 rpm for another 10 min. Then, 2 μL supernatant was subjected to UPLC-MS/MS analysis.

UPLC chromatograph coupled with a PerkinElmer QSight®210 MS/MS detector (PerkinElmer, Waltham, MA). The instrument control, analysis and data processing were performed using Simplicity 3Q™ software platform. Sample separation was achieved on an Acquity C_18_ column (4.6× 50 mm, 1.7 μm) with a constant flow rate of 0.3 mL/min at 30 °C. The mobile phase was composed of water (0.1% formic acid, A) and acetonitrile (C), using a gradient elution of 80-60% A at 0–4 min, 60-10% A at 4–6 min, 10-10% A at 6–7 min, 10-80% A at 7–8 min, 80-80% A at 8–10 min. The injected volume was set at 2 μL. The acquired parameters were optimized as follows: drying gas value, 100; nebulizer gas value, 150; electrospray voltage, 5500 V; HSID temperature, 280 °C. The detection was recorded as MRM negative mode. The proposed analytical method was validated and calculated for specificity, linearity, intra-day and inter-day precision, accuracy, extraction recovery, matrix effect and stability, according with the criteria described in the FDA guidelines for bioanalytical samples.

### Enzymatic assays

#### β-glycosidase

The assay for β-glycosidase activity was conducted according to the reported method with minor modifications [31]. Briefly, 100 μL fermented sample was acquired by centrifuging at 10,000 rpm for 10 min. The reaction mixture (1.0 mL) comprised of 1 mM p-nitropheny-β-D-glucopyranoside (PNP-D-Glu), 0.1 M phosphate buffer (pH 6.8) and the sample was incubated at 37 °C for 30 min. The reaction was stopped by adding 500 μL of 0.5 M NaOH centrifuged at 10,000 rpm for 10 min. The amount of PNP released was measured by absorbance at 405 nm in a microplate reader.

#### α-Glucosidase

The α-glucosidase inhibitory property was performed according to the previous method with modification [32]. The tested sample, diluted 5 times with water, was vortexed at 3000 rpm for 5 min. The reaction mixture was composed of the tested sample, phosphate buffer (0.1 M, pH 6.8) and α-glucosidase (50 μg/mL). Next, p-nitrophenyl-α-D-glucopyranoside solution (10 mM) was added to the mixture. The incubation was continued for 20 min at 37 °C, and which was stopped by adding 100 mM Na_2_CO_3_ solution. Acarbose was used as a positive control at 1 μg/mL. The reaction was measured by monitoring 405 nm. The results were presented as a percentage of α-glucosidase inhibition, calculated according to the following equation: ${\text{Inhibition }}\left( \% \right) \, = \, ({}{\text{OD}}{}\_\left( {{\text{ctrl}}.} \right) - {}{\text{OD}}{}\_{\text{sample}})/{}{\text{OD}}{}\_({\text{ctrl}}.) \, \times { 1}00\%$.

#### α-Amylase

The activity of α-amylase was measured using a modified method [33]. Briefly, the tested sample and α-amylase solution (0.2 U/mL) were incubated at 37 °C for 30 min. Next, 2% soluble starch solution was added to the mixture, and the incubation was continued for another 20 min at 37 °C. HCl (1 M) was added to terminate the enzymatic reaction, followed by iodine reagent (5 mg/mL). Acarbose (200 μg/mL) was used as a positive control. The absorbance was measured at 620 nm, and the percent of inhibition was calculated.

Pancreatic lipase: The pancreatic lipase activity was performed using PNPP as substrate [34]. PNPP was used as a substrate in a solution containing: 40 mg PNPP in isopropanol added to 50 mM Tris–HCl buffer (pH 8.0), 40 mg gum Arabic, 80 mg sodium deoxycholate, and Triton X-100. Orlistat (50 μg/mL) was used as a positive control. Briefly, 20 μL of the tested sample was put into 96-well plates, and lipase enzyme solution (10 mg/mL; porcine pancreatic lipase type II, Sigma-Aldrich) was freshly prepared in 50 mM Tris-HCl buffer (pH 8.0), stirred until fully dissolved and was then added 80 μL to all tests. After 37 °C for 15 min, the substrate solution was added at 37 °C for 25 min. Absorbance was recorded at 405 nm.

### Antioxidant activity

DPPH radical-scavenging capacity was estimated according to a previous protocol [35]. Vitamin C (100 μg/mL) was used as a positive control. Briefly, 80 μL of each tested sample and 800 μL DPPH (0.5 mmol/L) solubilized in a methanol solution were vortex-mixed and incubated in the dark at 37 °C for 20 min. DPPH radical was determined by measuring the absorbance at 517 nm. Total antioxidant capacity (T-AOC) was measured by biochemical methods following the manufacturer’s instructions (Beijing Solarbio Science and Technology, Beijing, China) [36].

### Anti-glycation assay

The lysine-glucose Maillard reaction was determined, as recorded previously [37]. Glutamic acid and lysine (both at 1.0 M, 0.2 mL) were mixed with 0.8 mL of tested samples in sodium phosphate buffer (0.1 M, pH 6.8) and 0.5 mL of 0.25 M sodium phosphate buffer at 70 °C for 2 h. Aminoguanidine (10 mg/mL) was used as a positive sample. The absorbance was measured at 450 nm on a microplate reader. The anti-glycation assay in the BSA-fructose model was performed as described [38]. Fructose (1.5 M, 0.5 mL) was mixed with 0.5 mL tested sample, 2.0 mL sodium phosphate buffer (0.1 M, pH 6.8, with 0.02% sodium benzoate.) at 37 °C for 2 h. BSA (30 mg/mL, 0.5 mL) was added at 37 °C for 5 days. Aminoguanidine (1 mg/mL) was used as a positive control. The fluorescent advanced glycation end-product (AGE) was monitored (350 nm as the excitation /420 nm as emission) using a fluorescence spectrophotometer. Methylglyoxal (60 mM, 0.5 mL) was mixed with 0.5 mL tested sample and 2.0 mL of sodium phosphate buffer (0.1 M, pH 6.8, with 0.02% sodium benzoate) at 37 °C for 2 h. BSA (30 mg/mL, 0.5 mL) was added at 37 °C for 5 days. Aminoguanidine (1 mg/mL) was used as a control. The fluorescent AGE was monitored (350 nm as the excitation /420 nm as emission) was measured on the fluorescence spectrophotometer. In arginine-methylglyoxal assay, methylglyoxal (60 mM, 0.5 mL) was mixed with 0.5 mL of tested samples and 2.0 mL sodium phosphate buffer (0.1 M, pH 6.8, with 0.02% sodium benzoate.) at 37 °C, 2 h. Arginine (60 mM, 0.5 mL) was added to all sets, and the mixtures were incubated at 37 °C for 5 days. Aminoguanidine (1 mg/mL) was used as a positive sample. Then, the fluorescent AGE was monitored (350 nm as the excitation /420 nm as emission) was measured.

### Methylglyoxal scavenging

Methylglyoxal scavenging was conducted by HPLC method according to a previously published method with modification [39]. Methylglyoxal was derivatized with O-phenylenediamine (O-PD) to form 2-methylquinoxaline (2-MQ), highly specific for methylglyoxal. Methylglyoxal and O-PD were dissolved in phosphate buffer (0.1 M, pH 6.8) to 10 and 50 mM. Aminoguanidine (1 mg/mL) was used as a control. The mixture of methylglyoxal (50 mM, 0.1 mL) with the sample (0.4 mL) was incubated at 37 °C for 4 h. Then, O-PD (0.2 mL) was added into all sets. The samples were kept for 30 min for undergoing derivatization reaction between methylglyoxal and O–PD. Analysis of 2-MQ was performed on a Waters 2695 HPLC platform (Waters Corporation, Milford, MA) and carried out by a Zorbax SB-C18 column (4.6 × 250 mm, 5 μm, Agilent Technologies, Palo Alto, CA). The mobile phase for HPLC system consisted of pure methanol (solvent A) and pure Millipore water (solvent B) with a constant flow rate set at 1.0 mL/min. An injection volume was 10 μL. The linear gradient for elution was: 0–35 min, 5–100% A; 35–45 min, 100–5%; followed by 5 min to re-equilibrate the system. 2-MQ was detected at 315 nm using a DAD detector having a retention time at 20.09 min. The peak area of 2-MQ in each sample was integrated. The methylglyoxal scavenging was calculated using the percentage (%) calculated from the homologous equation in BSA-fructose model.

## Results

### Method development and optimization

**Fig. 1:**
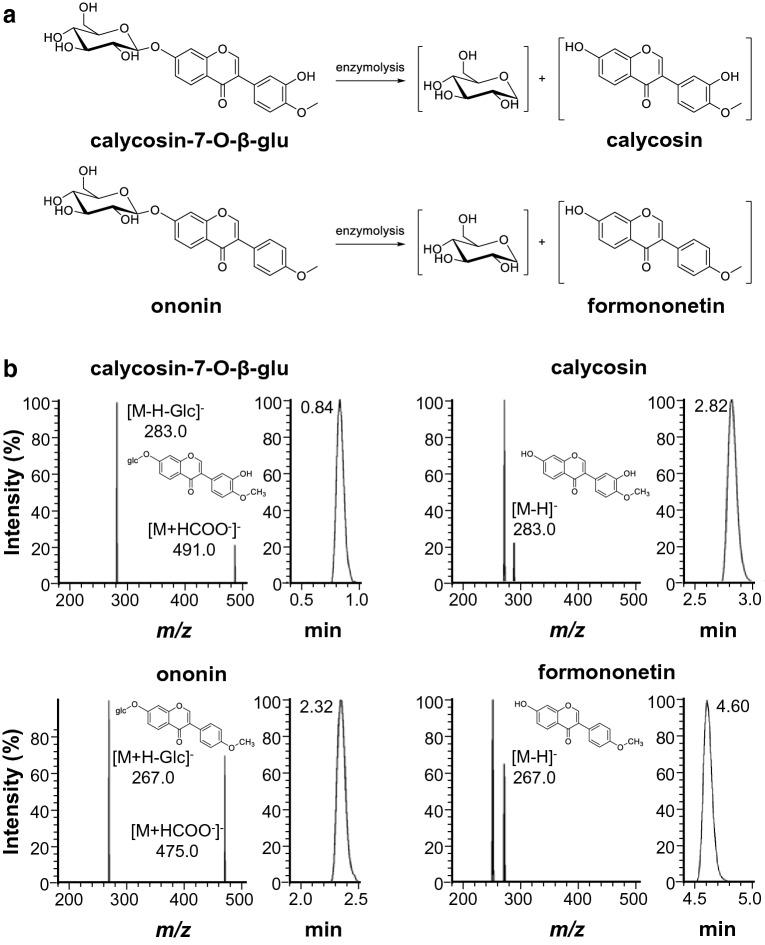
Conversion of flavonoid glycosides and its chemical analysis. **a** Conversion of calycosin-7-O-β-D-glucoside and ononin to calycosin and formononetin, examples of hydrolysis of flavonoid glycosides mediated by an enzymatic reaction. **b** Representative UPLC-MS/MS spectra of calycosin-7-O-β-D-glucoside, calycosin, ononin, and formononetin, showing precursor ion to product ion transitions, and their proposed fragmentation pathways

The chemical characterization of DBT was reported previously [8]. HPLC fingerprint of DBT, or fermented DBT, was shown for quality control (Additional file 1. Fig. S1). Besides, the amounts of major flavonoids in DBT, i.e. calycosin-7-O-β-D-glucoside, ononin, calycosin, formononetin, were subjected to UPLC-MS/MS analysis (Fig. 1a). Quantitative analysis of targeted analyte was performed by multiple reactions monitoring (MRM) in negative ion mode for better signal intensity. The parameters, including entrance voltage (EV), collision energy (CE) and collision cell lens (CCL), were optimized in accord to the peak intensity of analyte to minimize matrix effect, as well as to increase overall sensitivity. The optimized MRM parameters of the analytes, including IS (rutin), were shown in Additional file 1. Table S1. The deprotonated molecules [M-H]^−^ as precursor ions were shown for flavone aglycones, i.e. calycosin and formononetin. For the corresponding flavonoid glycosides, calycosin-7-O-β-D-glucoside and ononin, the anionic adducts [M + HCOO^−^]^−^ were detected as precursor ion, then HCOOH ion and sugar moiety could be lost from the precursor ion to form the deprotonated and de-glycosylated molecule [M-H-Glc]^−^. The UPLC chromatogram and related MS transition spectrum were presented in Fig. 1b.

**Fig. 2:**
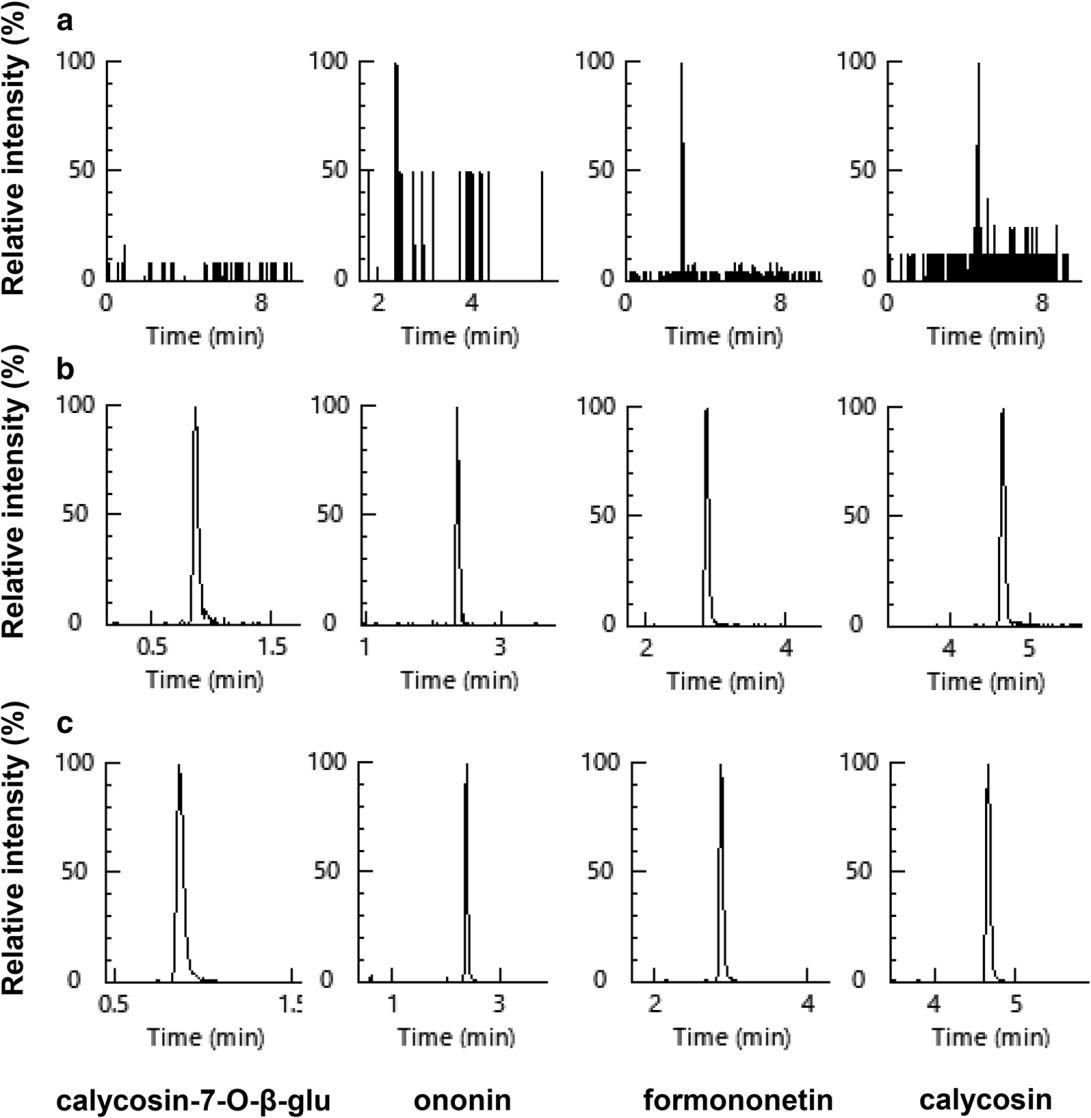
Chromatographic analysis of flavonoids. Representative chromatograms of calycosin-7-O-β-D-glucoside, ononin, formononetin and calycosin under **a** Blank matrix; **b** Blank matrix spiked with the four analytes LLOQ; and **c** The products at 6 h of fermentation

To validate the analytical method, a series of attributes were estimated, e.g. specificity, linearity, calibration range, sensitivity, precision, accuracy, extraction efficiency, matrix effect and stability. No interference of endogenous substance was found in the analytes at LLOQ, and all peaks possessed a good separation and dispersion (Fig. 2). The linearity of method for all analytes were investigated in the concentration range of 0.023—50 μg/mL, with the coefficient of determination (*R*^*2*^) greater than 0.99 (Table 1), which was acceptable for the quantification of samples. The sensitivity was evaluated by determination of LODs and LLOQs for each analyte (Table 1). Each analyte showed a LLOQ lower than 0.023 μg/mL. These findings suggested that the established MS method was sensitive enough to determine all analytes in the fermented broth.

**Table 1:** Linearity and sensitivity of UPLC-MS/MS assay for standards in fermented DBT

Analyte	Calibration curve	*R*^*2*^^a^	Linear range (μg/mL)	LLOQ (μg/mL)	LOD (μg/mL)
Cal-O-β-glu^d^	y = 0.1032x + 0.0397	0.9986	0.023–50	0.023	0.008
Ononin	y = 0.2496x + 0.1498	0.9967	0.023–50	0.023	0.008
Calycosin	y = 4.4401x + 4.0138	0.9909	0.023–50	0.023	0.008
Formononetin	y = 3.8437x + 3.2295	0.9923	0.023–50	0.023	0.008

^a^The calibration curve was constructed by plotting the peak area versus the concentration of each analyte. Each calibration curve was derived from 6 data points, *n* = 3. *R*^2^, coefficient of determination

*LLOQ* lower limit of quantification

*LOD* limit of detection

*Cal-O-β-glu*^d^ calycosin-7-O-β-D-glucoside

The intra- and inter- day assay performances were assessed by analyzing 3-level of QC samples (LQC, MQC and HQC) in six replicates, representing an entire calibrating range. The RE values of intra- and inter-day accuracies were in the range of -12.8% to 11.5% and -10.8% to 11.2%, respectively (Table 2). For intra- and inter-assay precisions, the RSDs were ranged from 0.84%—4.52% and 1.02%—8.51%, respectively. The above values were below 15%, demonstrating favorable data for acceptance criteria. The extracting efficiencies and matrix effects of analytes were determined in 6 replicates, and the results were summarized in Table 3. The extraction at three concentrations were satisfactory, as confirmed by recovery of 93.2–103.8%, indicating the acceptability and accuracy of this method. In terms of matrix effect, there was no significant matrix effect observed under current conditions, i.e. 89.6–114.3%, which was within the range of 80–120% [31]. In addition, the RSDs of extraction efficiencies and matrix effects for 3-level QC samples were below 15%, demonstrating a favorable sample preparation. The tests for assessing stability of established method were performed in auto-sampler at 4 °C for 48 h in a 8 h interval, having RSD values in a range of 1.48–5.81% and RE% of − 7.68–14.80% (Table 3). The results suggested the sample stability during storage.

**Table 2:** Intra-day and inter-day precision and accuracy for UPLC-MS/MS assay of standards in fermented DBT

Analyte	Spiked (μg/mL)	Intra-day (*n* = 6)^a^				Inter-day (*n* = 18)^b^		
		Measured (μg/mL)	Precision (RSD%)	Accuracy (RE%)		Measured (μg/mL)	Precision (RSD%)	Accuracy (RE%)
Cal-O-β-glu	0.07	0.07	4.52	2.77		0.07	4.09	4.24
	1.84	1.91	0.84	4.16		1.98	2.30	7.93
	16.60	16.30	2.04	− 1.97		16.70	1.74	− 0.14
Ononin	0.07	0.07	2.83	− 2.30		0.06	6.09	− 5.03
	1.84	2.03	1.92	10.20		2.09	2.48	13.90
	16.60	14.80	2.32	− 12.80		14.90	1.44	− 10.30
Calycosin	0.07	0.07	2.06	4.42		0.06	8.51	− 10.80
	1.84	2.05	1.32	11.50		2.04	2.56	11.20
	16.60	15.50	2.21	− 7.58		16.00	1.10	− 6.51
Formononetin	0.07	0.07	2.61	3.93		0.06	2.44	− 6.58
	1.84	1.99	1.91	7.99		1.99	2.63	7.88
	16.60	15.60	2.20	− 6.87		15.80	1.02	− 4.90

^a^The intra-day analysis refers to the sample examined for 6 replicates within one day

^b^The inter-day analysis refers to the sample examined in duplicates over 3 consecutive days

*Cal-O-β-glu* calycosin-7-O-β-D-glucoside

**Table 3:** Extraction efficiency, matrix effect and stability for UPLC-MS/MS assay of flavonoids in fermented DBT

Analyte	Spiked (μg/mL)	Extraction efficiency^a^ (*n* = 6)		Matrix effects^b^ (*n* = 6)		Autosampler stability^c^ (*n* = 6)	
		Measured (%)	RSD (%)	Measured (%)	RSD (%)	Accuracy (RE%)	RSD (%)
Cal-O-β-glu	0.07	101.20	2.49	114.30	8.18	4.29	11.50
	1.84	95.00	2.45	104.10	1.82	8.57	2.51
	16.60	99.20	1.82	110.20	1.13	1.33	1.48
Ononin	0.07	102.10	6.90	99.80	3.89	2.82	5.81
	1.84	93.30	2.95	97.20	2.61	14.80	1.95
	16.60	100.00	3.42	81.50	0.91	-7.68	2.41
Calycosin	0.07	103.80	1.76	99.50	2.36	-0.51	4.39
	1.84	93.30	2.43	98.20	1.10	12.30	2.82
	16.60	99.80	2.86	89.60	1.00	-7.28	3.29
Formononetin	0.07	100.10	1.62	100.30	3.21	15.50	3.76
	1.84	93.20	1.91	99.70	1.25	8.90	3.26
	16.60	99.70	2.68	90.50	0.95	-7.12	3.39

^a^Extraction efficiency (%) = 100% × amount found (pre-extraction) /amount found (after extraction). The data was presented as average of 6 independent determinations, and the SD was < 5% of the Mean, which was not shown for clarity

^b^Matrix effects (%) = 100% × amount found (after extraction)/amount spiked.The data was presented as average of six independent determinations, and the SD was < 5% of the Mean, which was not shown for clarity

^c^Autosampler stability (%) = 100% × amount found (pre extraction) − amount spiked/amount spiked. The data was presented as average of 6 independent determinations, and the SD was < 5% of the Mean, which was not shown for clarity

*Cal-O-β-glu* calycosin-7-O-β-D-glucoside

### Fermentation of DBT with *L. plantarum*

**Fig. 3:**
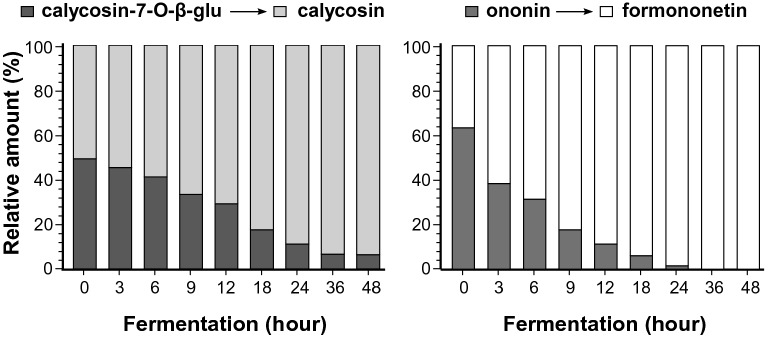
Hydrolysis of flavonoid glycosides during fermentation. The fermentation was started at time zero having *L. plantarum* 1 × 10^7^ CFU/mL with DBT herbal extract at 21.6 mg/mL in MRS at 37 °C for various time points, as indicated. The amounts of calycosin-7-O-β-D-glucoside, calycosin, ononin and formononetin were determined by UPLC-MS/MS as in Fig. 1. The relative amount of each flavonoid was shown. Values are in mean ± SD, *n* = 5. The SD is not shown for clarity

Probing the possible changes of DBT ingredients, *L. plantarum* was fermented in the presence of DBT for different periods. The validated method, as mentioned above, was applied to determine the concentrations of four flavonoids in fermented products. The parental DBT without fermentation, i.e. at time zero, contained calycosin-7-O-β-D- glucoside with its aglycon calycosin, as well as ononin with its aglycon formononetin (Fig. 3). The parental DBT contained calycosin-7-O-β-D-glucoside at 0.54 μg, ononin at 0.36 μg, calycosin 0.34 μg and formononetin at 0.12 μg per mg of dried herbal extract. According to the progression of fermentation, the amount of calycosin-7-O-β-D-glucoside decreased, and contrary the amount of calycosin was increased. This change was started to be significant after 12 h of fermentation. At 36 h of fermentation, the amount of calycosin-7-O-β-D-glucoside decreased to a minimum. This observation was revealed in a pair of ononin and formononetin. In contrast, the conversion was more robust in this scenario (Fig. 3). The completed conversion of ononin to formononetin was revealed after 24 h of fermentation. This conversion could be an outcome of enzymatic hydrolyzing of flavonoid glucosides to its aglycons.

**Fig. 4:**
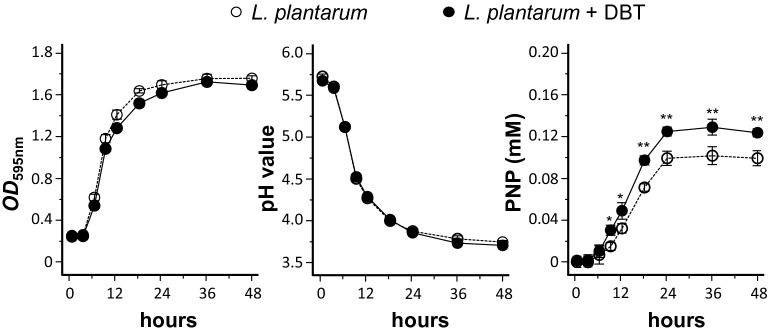
Growth of *L. plantarum* and activity of β-glucosidase in the presence of DBT. The fermentation was started at time zero having *L. plantarum* 1 × 10^7^ CFU/mL, or with DBT herbal extract at 43.2 mg/mL, in MRS at 37 °C for various time points, as indicated. The changes of growth (OD595 absorbance), pH value and release of p-nitrophenol (PNP, corresponding β-glucosidase activity) during the fermentation were measured in each time point. The fermented product (0.2 mL) was used for assayed of β-glucosidase. Values are represented as mean ± SD, *n* = 5. The significance difference was assessed by one-way ANOVA: *p < 0.05 and ** *p* < 0.01 vs. *L. plantarum* group (no DBT)

The growth (i.e. OD_595_ absorbance) and pH value of *L. plantarum* being fermented with or without DBT were determined. There was no apparent growth in the first 3 h, and however an exponential growth was observed thereafter (Fig. 4). The growth started to be a plateau at about 24 h of fermentation. In the presence of DBT or not, the growth of *L. plantarum* did not show much difference. The pH decrement was accompanied by culture growth during the whole fermentation process. No significant difference was observed for the two culture conditions (Fig. 4). Because of the conversion of flavonoid glycoside during fermentation, the enzymatic activity of β-glucosidase, an enzyme catalyzes the hydrolysis of the glycosidic bonds to terminal non-reducing residues in β-D-glucosides and oligosaccharides, was determined. The release of PNP from p-nitrophenyl-D-glucopyranoside (PNP-D-Glu), reflecting enzymatic activity β-glucosidase, was compared in the fermented product with or without DBT. In present of DBT, the release of PNP was increased markedly, reaching a plateau after 24 h of fermentation: this induction was significantly higher than that of *L. plantarum* without DBT (Fig. 4). This phenomenon suggested that the inclusion of DBT in fermentation could cause *L. plantarum* to produce higher amount of β-glycosidase in hydrolyzing flavonoid glycosides.

### Fermented product with DBT shows better anti-diabetic functions

**Fig. 5:**
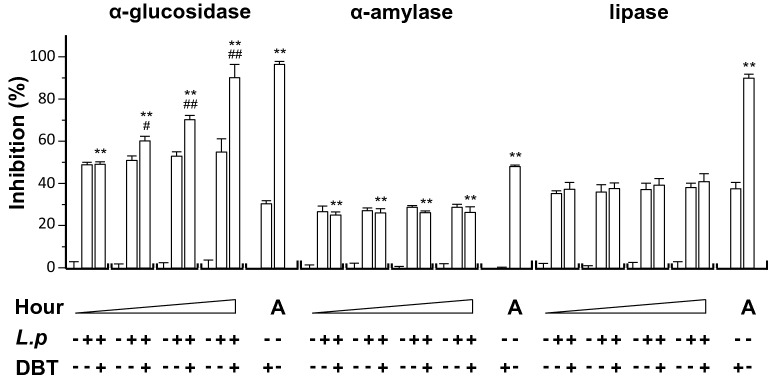
The fermented product having DBT shows stronger α-glucosidase activity. The effects of *L. plantarum* fermented DBT on α-glucosidase, α-amylase, and pancreatic lipase were measured. The fermentation medium was used as a control group. The fermentation was started with *L. plantarum* 1 × 10^7^ CFU/mL, or with DBT herbal extract at 43.2 mg/mL, in MRS at 37 °C for 0, 9, 18, and 48 h, as indicated. The products (0.2 mL) in all cases were used for assay. DBT dissolved in MRS at 43.2 mg/mL without *L. plantarum* and acarbose (A) served as positive controls for α-glucosidase (at 1 μg/mL) and α-amylase (at 200 μg/mL). Orlistat (O; 50 μg/mL) is a positive control. Values are represented as mean ± SD, *n* = 6, as a percentage of inhibition against control (no drug). The significance was assessed by one-way ANOVA: *p < 0.05 and **p < 0.01 vs DBT group, and ^#^*p* < 0.05 and ^##^*p* < 0.01 vs *L. plantarum* group (*L.p*)

The fermented products were tested for possible functions in diabetics. In contrast to β-glucosidase, α-glucosidase breaks down starch and disaccharides to glucose. Inhibitors of α-glucosidase, e.g. acarbose and voglibose, are common drugs for diabetes mellitus type 2, which are aiming to prevent the digestion of carbohydrate to simple sugar [33]. The usage of these inhibitors could lead to reduction of blood sugar. Here, DBT, fermented or not fermented, was tested for activity in inhibiting α-glucosidase. The culture medium of *L. plantarum* and DBT showed inhibition of about 30 to 50% (Fig. 5). After fermentation, the inhibition activity was markedly increased: the inhibition was close to ~ 90% after 48 h of fermentation. Without DBT, the fermented *L. plantarum* showed no increase activity at all. Acarbose served as a control showing inhibition, similar to that of fermented DBT. α-Amylase, an enzyme catalyzes the hydrolysis of starch into simple sugar, and lipase, an enzyme catalyzes hydrolysis of fat, were assayed for the DBT fermented products. DBT alone without fermentation showed low inhibition on the activities of α-amylase and lipase. Medium of *L. plantarum* showed inhibition at ~ 30%; however, the fermented DBT with *L. plantarum* did not change the pattern (Fig. 5). The positive controls of α-amylase (acarbose) and lipase (orlistat) showed significant inhibition (Fig. 5).

**Fig. 6:**
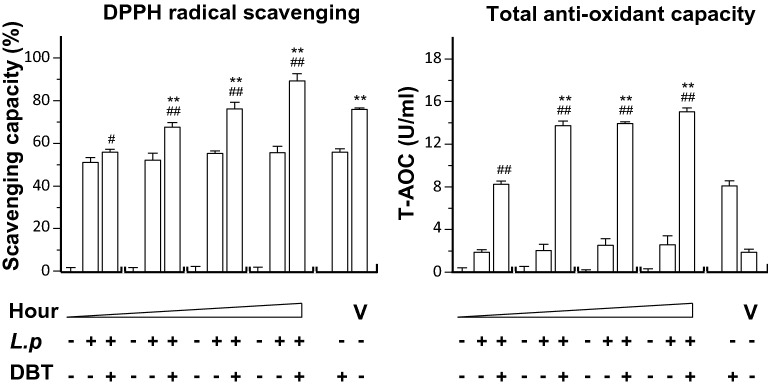
The fermented product having DBT shows stronger anti-oxidative activity. The effects of *L. plantarum* fermented DBT on DPPH radical scavenging and total antioxidant capacity (T-AOC) were measured. The fermentation medium was used as a control group. The fermentation was started with *L. plantarum* 1 × 10^7^ CFU/mL, or with DBT herbal extract at 43.2 mg/mL, in MRS at 37 °C for 0, 9, 18, and 48 h, as indicated. The products (0.2 mL) in all cases were used for assay. DBT dissolved in MRS at 43.2 mg/mL without *L. plantarum* and Vitamin C (V) served as positive controls at 100 μg/mL for DPPH scavenging and T-AOC). Values are represented as mean ± SD, *n* = 6, as a percentage of inhibition against control (no drug). The significance was assessed by one-way ANOVA: *p < 0.05 and **p < 0.01 vs DBT group, and ^#^*p* < 0.05 and ^##^*p* < 0.01 vs *L. plantarum* group (*L.p*)

The DPPH radical scavenging capacity of DBT, with or without fermentation, was measured. DBT alone showed DPPH radical scavenging, having ~ 50% reduction. Fermentation with *L. plantarum* could markedly enhance the scavenging activity of DBT: this increase was in a time-dependent manner (Fig. 6). Fermentation at 48 h increased the scavenging activity of DBT to almost 90%, better than the positive control (vitamin C). The total antioxidant capacity (T-AOC) was measured in DBT showing induction of T-AOC (Fig. 6). In a time-dependent manner, the fermentation of DBT with *L. plantarum* possessed robust activity of T-AOC, e.g. 15.0 U/mL of T-AOC after 48 h of fermentation.

**Fig. 7:**
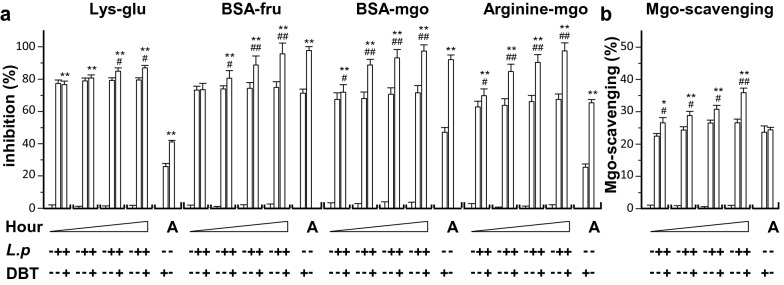
The fermented product having DBT shows stronger anti-glycation activity. The effects of *L. plantarum* fermented DBT on glycation of protein were measured, including **(A):** Lysine-glucose Maillard reaction (Lys-glu), BSA-fructose glycation model (BSA-fru), BSA-methylglyoxal glycation model (BSA-mgo), arginine-methylglyoxal glycation model (arginine-mgo) and **(B):** Mgo scavenging (Mgo-scavenging). The fermentation was started with *L. plantarum* 1 × 10^7^ CFU/mL, or with DBT herbal extract at 43.2 mg/mL, in MRS at 37 °C for 0, 9, 18, and 48 h, as indicated. The fermented products (0.2 mL) in all cases were used for assayed. DBT dissolved in MRS at 43.2 mg/mL without *L. plantarum* and aminoguanidine (A) served as a positive at 1 mg/mL, except for Lys-glu at 10 mg/mL. Values are represented as mean ± SD, *n* = 6, as percentage of inhibition against control (no drug).The significance was assessed by one-way ANOVA: *p < 0.05 and **p < 0.01 vs DBT group, and ^#^*p* < 0.05 and ^##^*p* < 0.01 vs *L. plantarum* group (*L.p*)

Glycation is a reaction of free reducing sugars with free amino groups of protein, DNA and lipid forming Amadori products, subsequently which leads to the formation of advanced glycation end product (AGE). This process leads to a loss of protein function and impaired cell functions. The glycation reaction is highly accelerated under hyperglycemia, implicating the pathogenesis of diabetics and aging. In addition, oxidative stress plays a role in forming AGEs, and thus which has been implicated in causing the progression of diabetes [34]. In view of this, the possible functions of anti-glycation of DBT with or without *L. plantarum* fermentation were determined here. In Maillard reaction, lysine-glucose was detected as brown color, revealed under absorbance at 450 nm, after heating at 70 °C for 2 h. DBT, or *L. plantarum* medium, showed the browning inhibition, and the inclusion of two of them did not show increase of inhibition (Fig. 7). The fermentation of DBT however could increase the browning inhibition, reaching ~ 80% of inhibition. The BSA-fructose model was used here to further demonstrate the anti-glycation of DBT. The fluorescent AGE was monitored using a fluorescence spectrophotometer (350 nm as excitation /420 nm as emission). DBT itself showed glycation inhibition, and which was robustly enhanced by fermented together with *L. plantarum* (Fig. 7). This enhancement was in a time-dependent manner.

The BSA-methylglyoxal model represents the middle stage of protein glycation, leading to formation of AGE. From reading of fluorescent AGE, the fermentation of DBT displayed higher anti-glycation activity in a time-dependent manner (Fig. 7). In arginine-methylglyoxal model, the formation of AGE was suppressed by DBT, and which was markedly enhanced by fermentation: the maximal inhibition was close to a completion (Fig. 7). Similar to the aforementioned anti-glycation activities, DBT was able to inhibit the scavenging activity, and the activity was markedly enhanced after fermentation with *L. plantarum* (Fig. 7). Aminoguanidine served as a positive control here showing inhibition in all cases.

## Discussion

Fermentation of *L. plantarum* together with a Chinese herbal mixture DBT could enhance the bacterial growth, as well as the chemical transformation of herbal extract. In chemical transformation, the major flavonoid glucosides of DBT were hydrolyzed into their corresponding aglycones: this conversion was mediated by β-glucosidase deriving from fermented bacteria. For the first time, we have utilized *L. plantarum* bacteria in strengthen the pharmacological efficacy of DBT. The fermented DBT showed better effects in anti-diabetic functions, which included: (i) inhibitory properties on α-glucosidase; (ii) antioxidant properties on DPPH scavenging and T-AOC; and (iii) anti-glycation capacity on various models. This fermentation approach, nevertheless, could be considered as a mean to enhance the pharmacological efficacy of TCM in general. The safety should not be a concern: because *L. plantarum* is a common *Lactobacillus* found in many fermented food products, as well as in human saliva, which has been known to show no harm to our body. Having this new direction of fermentation of Chinese herbal extract therefore can pave another method to enhance the product efficacy, in particular *Lactobacillus* and herbal product are commonly used as health food products on the market.

The UPLC-MS/MS analysis is a common method for simultaneous quantitation of flavonoid compounds. However, most of the developed methods are focusing on herbal or plasma samples. No reports have investigated the simultaneously quantitative analysis of the flavonoids in the fermented broth of *L. plantarum*. The method development of MS parameters, as well as method validation comprising parameters, e.g. specificity, linearity, sensitivity, precision and accuracy, extract efficiency, matrix effects and stability, is being described in detail at the present study. Thus, a sensitive and reliable quality control method using MRM in negative ion mode for simultaneous determination of four flavonoids in fermented DBT, i.e. calycosin-7-O-β-D-glucoside, ononin, calycosin and formononetin, has been developed.

The chemical transformation, i.e. cleavage of the sugar moieties, was observed in here. The enzyme β-D-glucosidase is responsible for the cleavage of β-D-glycosidic linkages, releasing glucose moieties from flavonoid glycosides [39]. This enzyme should derive from the cultured medium of *L. plantarum*, not from the herbal extract. The expression of β-D-glucosidase in the fermented product, as revealed by release of p-nitrophenol from p-nitropheny-β-D-glucopyranoside, could be markedly enhanced in DBT extract. A synergy during the fermentation of DBT with *L. plantarum* is being proposed here. First, DBT promoted the expression of β-D-glucosidase in fermenting *L. plantarum.* Second, the produced β-D-glucosidase hydrolyzed the flavonoid glycosides of DBT to aglycons. These findings suggested that an improvement in hydrolysis rate of glycoside enzymes perhaps may be ascribed to DBT inducement. In TCM preparation, the phytochemicals exhibit low oral bioavailability, in particular glycosides are possessing poor membrane permeability [40]. Moreover, these glycosides are inevitable to be transformed by microbial hydrolysis in intestinal before their absorption to the gut [41]. Herein, the role of β-D-glucosidase in food and/or pharmaceutical bioprocessing could promote aglycone entering blood circulation [42].

The anti-diabetic functions of fermented products should be derived from DBT. The chemical changes within DBT after fermentation could account for increased pharmacological efficacy. However, the identity of active chemicals of DBT in triggering these activities are not resolved. The increased antioxidant property could be accounted for the hydrolyzed flavonoid glycosides. Indeed, aglycons in the herbal extract were shown to have stronger antioxidative activity [43]. Although we have shown the increased calycosin and formononetin being generated from calycosin-7-O-β-D-glucoside and ononin, respectively, in the fermented DBT, the amounts of total aglycons should be more than that, i.e. other flavonoid glycosides in DBT should be undergone the transformation. In our preliminary results of the four AR flavonoids, the increased activities however could not be fully accounted by completed hydrolyzed of calycosin-7-O-β-D-glucoside and ononin. The inhibitory properties of α-glucosidase and glycation increased in the fermented DBT. The α-glucosidase inhibitory activity from natural products has been proposed to be accounted by flavone, in particular isoflavone [44]. In line to our current result of chemical conversion, flavonoid glucosides possessed a relatively poor inhibitory action on α-glucosidase, as compared with their corresponding aglycones [40].

The non-enzymatic glycation, a spontaneous reaction between sugar and protein, is considered as a source of oxidative stress, as well as the primary route in forming AGEs. Increased AGE has been implicated in the pathogenesis of diabetic complication. The increased anti-glycation activity in fermented DBT could be accounted by the conversion of flavonoid glucosides to aglycons. Various phenolic compounds, inhibiting α-glucosidase, have been reported as inhibitors of glycoside hydrolase due to their binding with proteins, as such to erode the glycation reaction [38]. The hydrolysis of polysaccharides within DBT during fermentation could be another cause of the increased anti-glycation activity; however, this notion has to be further illustrated.

## Conclusions

To the best of our knowledge, we have reported for the first time, the conversion of calycosin-7-O-β-D-glucoside and ononin to their aglycons in DBT through a fermentation process using *L. plantarum*. In considering the traditionally oral intake of herbal medicine, the conversion of flavonoid glycosides in fermented DBT not only improves the absorptivity of flavonoids to gut, but which possess prominent activities against α-glucosidase, antioxidant and anti-glycation. These anti-diabetic activities could be accounted, at least partially, by the increased flavonoid aglycons in the fermented products. The result paves direction for fermentation of herbal extract, as to strengthen its pharmacological properties.

## Supplementary information

**Additional file 1:** **Figure S1.** Typical-HPLC fingerprints of DBT before and after fermentation. **Table S1.** Analytic parameters of 4 standards by UPLC-MS/MS in fermented DBT.

## Data Availability

The datasets used and/or analyzed during the current study are available from the corresponding author on reasonable request.

## Abbreviations

AGE: The fluorescent advanced glycation end-product
ASR: Angelicae Sinensis Radix
AR: Astragali Radix
BSA: Bovine serum albumin
CCL: Collision cell lens
CE: Collision energy
Ctrl.: Control
DBT: Danggui Buxue Tang
EV: Entrance voltage
HQC: High quality control
IS: Internal standard
LLOQ: Lower limit of quantification
*L. plantarum*: *Lactobacillus plantarum*
LOD: Limit of detection
LQC: Low quality control
2-MQ: 2-Methylquinoxaline
MQC: Middle quality control
MRM: Multiple reactions monitoring
MRS: De Man, Rogosa, Sharpe
OD: Optical density
O-PD: O-phenylenediamine
PNP: P-nitrophenol
PNP-D-Glu: P-nitropheny-β-D-glucopyranoside
PNPP: P-nitrophenylpalmitate
QC: Quality control
RE: Relative error
RSD: Relative standard deviation
T-AOC: Total antioxidant capacity
TCM: Traditional Chinese medicine
T2DM: Type 2 diabetes mellitus
