# Supplementary material for: Fermentation of Danggui Buxue Tang, an ancient Chinese herbal mixture, together with Lactobacillus plantarum enhances the anti-diabetic functions of herbal product
Source: Chin Med. 2020 Sep 11;15:98. doi: 10.1186/s13020-020-00379-x (PMC7488747; doi:10.1186/s13020-020-00379-x)
Supplement: Supplementary file 1 — Additional file 1: Figure S1. Typical-HPLC fingerprints of DBT before and after fermentation. Table S1. Analytic parameters of 4 standards by UPLC-MS/MS in fermented DBT. [file 13020_2020_379_MOESM1_ESM.pdf]

Fermentation of Danggui Buxue Tang, an ancient Chinese herbal mixture, together with *Lactobacillus plantarum* enhances the anti-diabetic functions of herbal product

Rui Guo [1,2,†] , Shuchen Guo [1,†], Xiong Gao [1,3], Huaiyou Wang [1], Weihui Hu [1,3], Ran Duan [1,3], Tina T. X. Dong [1,3], Karl W. K. Tsim [1,3,*]

[1] Shenzhen Key Laboratory of Edible and Medicinal Bioresources, HKUST Shenzhen Research Institute, Shenzhen 518057, China
[2] Key Laboratory of Innovative Drug for the Treatment of Serious Diseases Basing on the Chronic Inflammation, Shanxi University of Chinese Medicine, 121 Daxue Road, Yuci District, Jinzhong 030619, China
[3] Division of Life Science and Center for Chinese Medicine, The Hong Kong University of Science and Technology, Hong Kong, China

*Corresponding author at Shenzhen Key Laboratory of Edible and Medicinal Bioresources, HKUST Shenzhen Research Institute, Shenzhen 518057, China; E-mail addresses: botsim@ust.hk (Karl W. K. Tsim)

[†] Rui Guo and Shuchen Guo contribute equally to this work and joint first authors.

Received: date; Accepted: date; Published: date

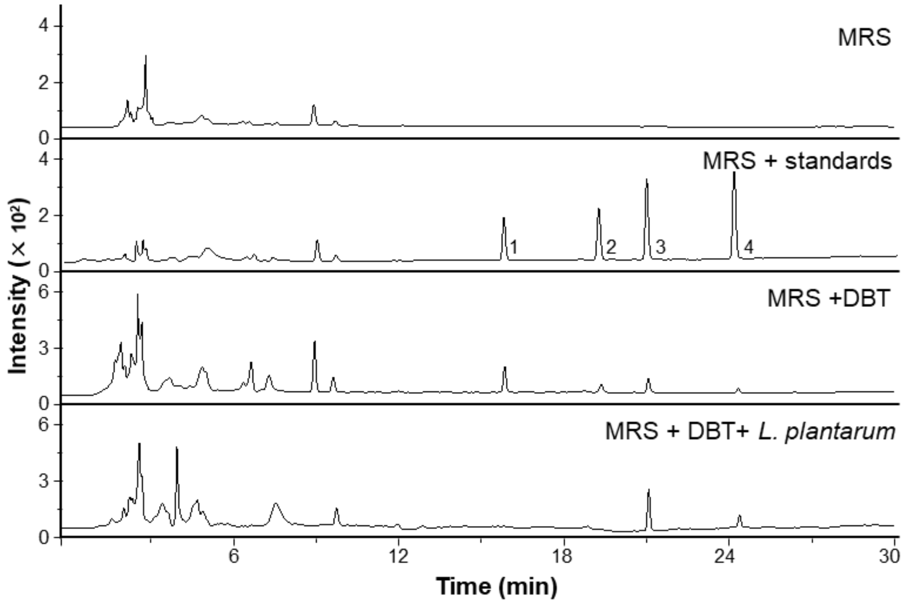

Figure 1S: Typical-HPLC fingerprints of DBT before and after fermentation. Absorbance at 254 nm was detected here. MRS is the background signal. Calycosin-7-O-β-D-glucoside (1), ononin (2), calycosin (3), and formononetin (4) are indicated with 20 μg/mL each. DBT (21.6 mg/mL) in MRS are shown. Fermentation of DBT (21.6 mg/mL) with *L. plantarum* (1×10^7 CFU/mL) in MRS was at 37 °C for 48 hours.

Supplementary Table 1S: Analytic parameters of 4 standards by UPLC-MS/MS in fermented DBT.

No.	Rt (min)	Analyte	Mw	Precursor ion[a]	Product ion[b]	Entrance voltage (V)	Collision energy (eV)[c]
1	0.85	Cal-O-β-glu[d]	446.40	491	283	-30	-22
2	2.35	Ononin	430.40	475	267	-30	-22
3	2.84	Calycosin	284.26	283	268	-30	-25
4	4.64	Formononetin	268.27	267	252	-30	-28
IS	0.76	Rutin	610.52	609	301	-30	-46

[a] The detected chemicals had the greatest responses under the negative mode: the $[M\text{-}H]^-$ or $[M+HCOO^-]^-$ were used as the precursor ion.

[d] Two product ions were used for the MRM analysis. The upper one was used for quantitative analysis and the lower one was for qualitative analysis, which could guarantee the precision of analytes.

[c] The collision energy was optimized to have the greatest product ion intensity, which was the key factor in the MRM mode.

[d] Cal-O-β-glu: calycosin-7-O-β-D-glucoside.
